# Identifying Age Cohorts Responsible for Peste Des Petits Ruminants Virus Transmission among Sheep, Goats, and Cattle in Northern Tanzania

**DOI:** 10.3390/v12020186

**Published:** 2020-02-07

**Authors:** C. M. Herzog, W. A. de Glanville, B. J. Willett, I. M. Cattadori, V. Kapur, P. J. Hudson, J. Buza, E. S. Swai, S. Cleaveland, O. N. Bjørnstad

**Affiliations:** 1 Center for Infectious Disease Dynamics, Pennsylvania State University, University Park, State College, PA 16802, USA; imc3@psu.edu (I.M.C.); vkapur@psu.edu (V.K.); pjh18@psu.edu (P.J.H.); onb1@psu.edu (O.N.B.); 2 Institute of Biodiversity, Animal Health and Comparative Medicine, University of Glasgow, Glasgow G12 8QQ, UK; Will.deGlanville@glasgow.ac.uk (W.A.d.G.); Sarah.Cleaveland@glasgow.ac.uk (S.C.); 3 MRC-University of Glasgow Centre for Virus Research, University of Glasgow, Glasgow G61 1QH, UK; Brian.Willett@glasgow.ac.uk; 4 Nelson Mandela African Institute of Science and Technology, Arusha, P.O. Box 447, Tanzania; joram.buza@nm-aist.ac.tz; 5 Department of Veterinary Services, Ministry of Livestock and Fisheries, Dodoma, P.O. Box 2870, Tanzania; esswai@gmail.com

**Keywords:** Epidemiology, peste-des-petits-ruminants, seroepidemiologic studies, Tanzania, force of infection, catalytic model

## Abstract

Peste des petits ruminants virus (PPRV) causes a contagious disease of high morbidity and mortality in global sheep and goat populations. To better control this disease and inform eradication strategies, an improved understanding of how PPRV transmission risk varies by age is needed. Our study used a piece-wise catalytic model to estimate the age-specific force of infection (FOI, per capita infection rate of susceptible hosts) among sheep, goats, and cattle from a cross-sectional serosurvey dataset collected in 2016 in Tanzania. Apparent seroprevalence increased with age, reaching 53.6%, 46.8%, and 11.6% (true seroprevalence: 52.7%, 52.8%, 39.2%) for sheep, goats, and cattle, respectively. Seroprevalence was significantly higher among pastoral animals than agropastoral animals across all ages, with pastoral sheep and goat seroprevalence approaching 70% and 80%, respectively, suggesting pastoral endemicity. The best fitting piece-wise catalytic models merged age groups: two for sheep, three for goats, and four for cattle. The signal of these age heterogeneities were weak, except for a significant FOI peak among 2.5–3.5-year-old pastoral cattle. The subtle age-specific heterogeneities identified in this study suggest that targeting control efforts by age may not be as effective as targeting by other risk factors, such as production system type. Further research should investigate how specific husbandry practices affect PPRV transmission.

## 1. Introduction

Peste des petits ruminants virus (PPRV), or small ruminant morbillivirus (SRMV), is a socio-economically important, highly infectious virus that causes high morbidity and mortality among sheep and goat populations worldwide. PPRV is present in Africa, the Middle East, and Asia, as well as in the country of Turkey, and currently impacts 80% of the world’s sheep and goat population [1], causing an estimated $1.45–2.1 billion USD in global annual losses due to mortality, impaired production, and treatment of infected animals [1]. Livestock keepers in these regions rely heavily on sheep and goats for their livelihoods, as they are a source of meat, milk, and income. Household herd losses due to PPRV contribute to global poverty and food insecurity. In 2015, the Food and Agricultural Organization (FAO) and World Animal Health Organization (OIE) launched a global campaign to eradicate PPRV by 2030. Although the available, affordable vaccine has demonstrated protection for up to 3 years [2], the large, global small ruminant population turns over rapidly, increasing the difficulty of implementing control strategies, such as blanket mass vaccination. To better target eradication control efforts, FAO also launched a global PPRV research network in 2018 [3] with the goal of aligning research efforts to inform strategies for PPRV eradication. One such research effort highlighted was the need to determine how PPRV transmission patterns vary by age, including identifying the appropriate age cohorts at which PPRV vaccination can be performed efficaciously and the age at which maternal immunity falls beneath a protective threshold (and what the value of the protective threshold is) [4].

The role of age in vulnerability to disease transmission is an important consideration that often leads public health and animal health experts to focus their control efforts on specific subsets of populations [5]. For many well-known infectious diseases, transmission risk varies by age. For example, measles, mumps, and rubella became known as childhood diseases in the pre-vaccine era as the force of infection (FOI, per capita rate of infection of susceptible hosts) was greatest during early childhood [6,7] due to age-stratified mixing and an adult population protected from previous exposure. Sexually transmitted infections typically have higher incidence and FOI after the age of sexual maturity among more sexually active individuals. Other infections, including influenza or bacterial infections, may impact any age group but have a greater disease impact among those who are immunocompromised—often the youngest and oldest members of a population [8]. In animals, evidence of age-dependent patterns in susceptibility and transmission have been observed for feline leukemia virus in cats and bovine tuberculosis in bison [9,10,11]. In these infections, young animals are more susceptible to infection, and seropositivity increases in the population with age. These age-related infection patterns are influenced by mixing patterns, which are likely to be different among wild animals, domesticated animals in different production systems, and children, who experience age-stratified mixing at school. Nevertheless, given that age-related patterns are seen among both human and animal diseases, calculating the FOI across age cohorts is a useful approach that can reveal which age group is responsible for most of the onward transmission. Prevention and control efforts can then be targeted to the appropriate age cohorts, thereby reducing transmission and lowering control costs compared with mass interventions, assuming the cost of identifying target groups is not prohibitive [12].

PPRV research to date has demonstrated that young animals may be protected for the first 3–5 months of life by maternal immunity [13,14,15,16,17], but once that protection wanes, young animals appear to be the most severely affected age group in terms of susceptibility and mortality [16,17,18]. The majority of published studies (compiled in Text S1) report age as a significant risk factor for PPRV seroconversion and that PPRV seroprevalence increases with animal age; however, there are also several studies that concluded age was not a significant risk factor. Further clarification is needed regarding the impact of age on PPRV transmission. 

**Figure 1:**
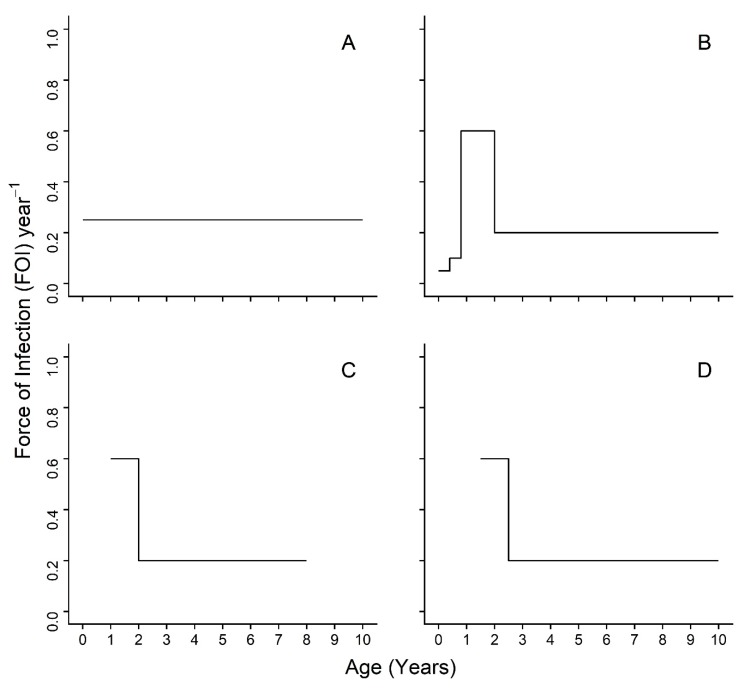
Schematic representation of the possible age-specific force of infection transmission patterns for Peste des petits ruminants virus (PPRV). Possible transmission routes include (**A**) constant force of infection from birth due to environmental exposure and (**B**) horizontal transmission during routine social activities at the age of independence and then at the time of breeding; (**C**) the expected patterns among the sheep and goats in the five age group dataset dominated by horizontal transmission due to breeding; (**D**) the expected pattern among cattle in the five age group dataset dominated by horizontal transmission due to socialization at age of independence.

To understand the role of age in PPRV transmission and to inform strategies for PPR eradication by vaccination, this study aims to identify which age cohorts are responsible for PPRV transmission using cross-sectional serosurvey data of sheep, goats, and cattle managed together in the same households under differing production systems across 20 villages in northern Tanzania. Earlier work on this population [19] did not assess age variation in transmission. We predict that seroprevalence will increase by age group for each species, as would be expected if PPRV were an endemic, fully-immunizing (i.e., providing life-long protection) infection. We use a piece-wise catalytic model framework to calculate age-varying FOI estimates. Our a priori set of hypotheses for the piece-wise catalytic model are represented schematically in Figure 1 using arbitrary FOI values for the step function. We postulate that an age-varying model (Figure 1B–D) will be a better fit to serosurvey data than an age constant model (Figure 1A). Furthermore, we predict that the highest FOI estimates will appear in the youngest age groups, shortly after the waning of maternal immunity, which is consistent with the hypothesis of horizontal transmission by socialization at the age of weaning and independence around a few months of age, or, in the case of sheep and goats, reproduction starting shortly before one year of age (Figure 1B). It will be possible to detect, but not separate, these two events in sheep and goats, as animals in the data set are all six months or older and the dentition-based age groups do not provide fine enough age resolution. As a precaution, models without the first age group were investigated in case of extended maternal immunity beyond the published estimates. The hypothesized transmission patterns for these models are presented in Figure 1C,D and include abbreviated FOI peaks for the youngest age groups.

## 2. Methods

Blood samples were collected in 2016 from clinically healthy sheep (*Ovis aries*), goats (*Capra aegagrus hircus*), and cattle (*Bos taurus indicus*) in 20 northern Tanzanian villages in Arusha and Manyara Regions as part of the “Social, Economic, and Environmental Drivers of Zoonoses” (SEEDZ) study. The SEEDZ study data collection, data cleaning, and laboratory testing methods have been described in detail previously [19]. The relevant methods have been summarized here. Briefly, a total of 7576 animals from 417 households were studied, and 404 household surveys were conducted using multistage random sampling. These households were from villages classified by livelihood type as either ‘pastoral’ (P) villages (those in which livestock rearing was considered the primary livelihood activity) or ‘agropastoral’ (AP) (those villages in which a mix of crops and livestock were important livelihood activities). A maximum of 10 cattle, 10 sheep, and 10 goats per selected household were randomly selected for sampling after restraint. Among the cattle, a target of five animals between 6 and 18 months of age and five animals over 18 months of age were selected. Among the sheep and goats, a target of five animals between 6 and 12 months of age and five animals over 12 months were selected. At least two of the animals were male in each of the groups, and all animals were old enough to be expected to lack maternally derived PPRV immunity [13]. Most animals (7122, 95%) were indigenous breeds; the rest were crossbreed (370, 4.9%) or exotic (4, 0.05%) [19].

Animal age was assessed by dentition, namely incisor tooth eruption and wear [20,21,22]. This age estimation method was used because the exact recorded ages were not available. Among samples containing complete species, sex, age, and location data, a total of 7538 serum samples were tested in duplicate using a commercially available competitive ELISA kit (Pirbright Institute, Surrey, England) directed against the hemagglutinin protein of PPRV [23,24]. A minority of samples were in disagreement and were tested a second time and the positive/negative status was resolved for all samples. Samples were heat inactivated (56 °C, 2 h) prior to shipment to the University of Glasgow for testing. Forty-two samples (0.6% of total) were removed from analysis as they were from households that self-reported PPRV vaccination in the past 24 months. However, 427 samples (5.7% of total) were retained in the analysis despite lacking a household survey from which to discern self-reported PPRV vaccination, as an exploratory analysis that excluded and included samples yielded qualitatively and quantitatively similar results. As previously reported [19], to our knowledge, government-led PPRV vaccination campaigns ceased as of 2013, and rinderpest virus vaccination ceased in Tanzania in 1997–1998, so cross-reactivity on the PPRV cELISA kit in this study was not a concern. Our final analysis sample included 7496 animals (2080 sheep, 2419 goats, 2997 cattle).

To understand the role of age heterogeneities in PPRV transmission, we calculated the force of infection (FOI), the rate of infection of susceptible hosts, using the catalytic framework [25]. The catalytic model provides the framework for calculating the FOI from age-specific seroprevalence data from cross-sectional surveys [25,26,27]. Given that the rate at which immunity builds up with age depends on rates of circulation, this approach allows us both to elucidate the rate of circulation (FOI, *λ*) and to determine which age classes are most important for continued transmission. According to the catalytic model, age-specific seroprevalence, *P*(*a*), can be expressed as

(1) $$\frac{dP\left(a\right)}{da}=\;\lambda \left(a\right)\left(1-P\left(a\right)\right)$$

where (1 − *P*(*a*)) is the proportion of susceptible hosts of age a, and *λ*(*a*) is the age-specific FOI. Integrating and rearranging yields:

(2) $$P\left(a\right)=1-exp\left[-\int_{0}^{a}\lambda \left(a\right)da\right]$$

which describes how the cumulative FOI up to a given age will deplete a susceptible cohort, where *P*(*a*) represents the probability of having been infected before age *a*. Age-specific seroprevalence curves are direct empirical observations on this probability. This model assumes endemicity and that seronegatives are fully susceptible.

In this study, age–seroprevalence curves were used to describe the apparent and true (adjusted) seroprevalence patterns across ages. Adjustment was conducted according to the method of Rogan and Gladen 1978 [28], using recalculated sensitivity and specificity estimates from Couacy-Hymann et al. [24], alongside our current in-house estimates of specificity of >99% for all three species, as well as sensitivities of 88%, 81%, and 48% in sheep, goats, and cattle, respectively, when cELISA was compared to a gold standard virus neutralization-based test. Power analysis indicated that a minimum sample size of 15 animals was needed in any age group to detect a 10% difference (medium effect size) between groups at power ≥80%. All stratifications except male sheep in age group 6 reached this criterion, and a two-proportion significance test was used to identify significant differences at a 5% level. 

The piece-wise catalytic model (2) was used to calculate age-specific FOI by species, species and sex, and species and management system. This model assumes a fixed FOI within the specified age intervals (dentition-based age groups converted into age in years). The age intervals were defined by the upper age cutoff: 1, 1.5, 2, 3, 5, and 8 years for sheep and goats [20,21] and 1.5, 2.5, 3.5, 4.5, 7, and 10 years for cattle [22]. All individual animal ages were set to the midpoint of the age interval (e.g., an animal in dentition-based age group 1, with an age interval of 0 to 1 years, would have its age reset to 0.5 years).

The model was optimized in a three-step process. Arbitrary initial parameter values (0.001) and a user defined function containing the model evaluated using exact integration were provided to the R software optim function. Parameter estimates were obtained using the quasi-Newton Broyden–Fletcher–Goldfarb–Shanno (BFGS) algorithm to minimize the negative log-likelihood. The first estimates were provided as initial values for a second optimization using the BGFS algorithm, and then the second estimates were used during a third optimization using the derivative free, stochastic, global optimization algorithm simulated annealing (SANN). Standard errors were computed using partial profile likelihood, following the method presented in Long et al. [29]. Although all animals were six months or older and not expected to have maternal immunity [13], models with and without the first age interval (<1-year-old, temporary teeth) were explored to demonstrate the impact on the FOI estimates. A model with five age intervals (first age interval removed) was preferred (presented in Figure S2, Tables S8–10) over the six-age interval model (presented in Figure S1, Tables S5–S7), due to unrealistically low estimates of the second age interval. Additionally, nested models were used to understand the impact of different combinations of merged, neighboring age intervals on model fit. The best fitting model for each species was selected by Akaike Information Criterion [30] and are presented in the text. All analyses were conducted in R software, version 3.5.3 [31].

### Ethics Statement

All adult participants in the SEEDZ study provided written informed consent. No data were collected from minors or children. In this study, questionnaire data were used solely to determine the PPRV vaccination status of household herds in the past 12 months. The protocols, questionnaire, and consent procedures were approved by the following ethical review committees: Kilimanjaro Christian Medical Centre (KCMC/832, issued 27 May 2015, renewed to 16 May 2020) and the National Institute of Medical Research (NIMR/HQ/R.8a/Vol.IX/2028, approved 7 October 2015, extended until 10 September 2020 by NIMR/HQ/R.8c/Vol.1/1328, approved 30 September 2019 ) in Tanzania; and the College of Medical, Veterinary, and Life Sciences, University of Glasgow in the United Kingdom (2001401521, approved 1 July 2015). Approval for animal work was provided by the Clinical Research Ethics Committee at the University of Glasgow School of Veterinary Medicine (39a/15, approved 15 October 2015), which authorizes research under The Veterinary Surgeons Act UK 1996 and oversees research regulated under the Animal (Scientific Procedures) Act 1986. Permission to publish this work was granted by the Director of Veterinary Services, Tanzania.

## 3. Results

**Figure 2:**
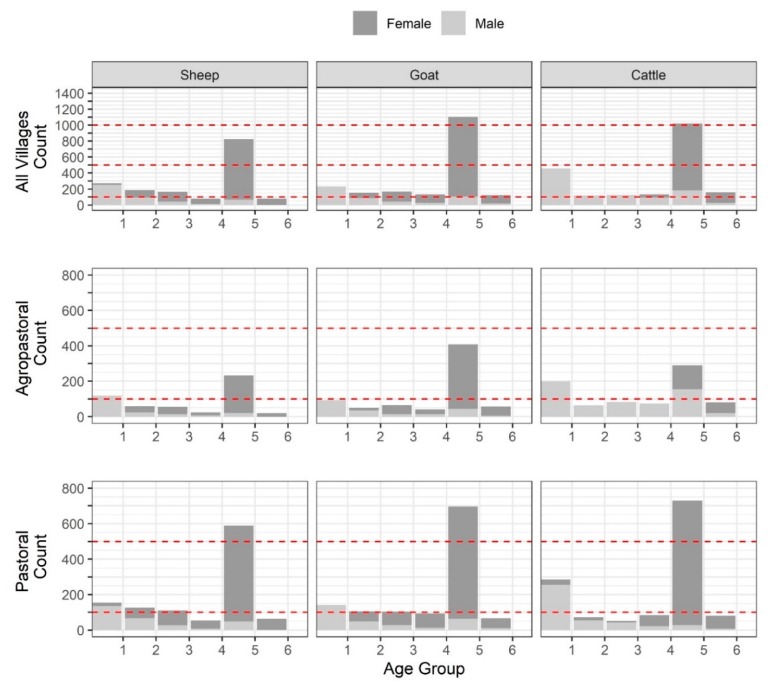
Dentition-based age group distribution by species, sex, and management system.

Demographic characteristics of the 7496 samples analyzed were published previously [19], a subset of which are included in Table S1. Overall, there were 1869 (24.9%), 757 (10.1%), 666 (8.9%), 483 (6.4%), 3307 (44.1%), and 414 (5.5%) animals in age groups 1–6, respectively (Figure 2, Table S2). For each species, the largest number of sampled animals was found in the fifth age group (full mouth with no wear), followed by the first age group (temporary teeth).

**Figure 3:**
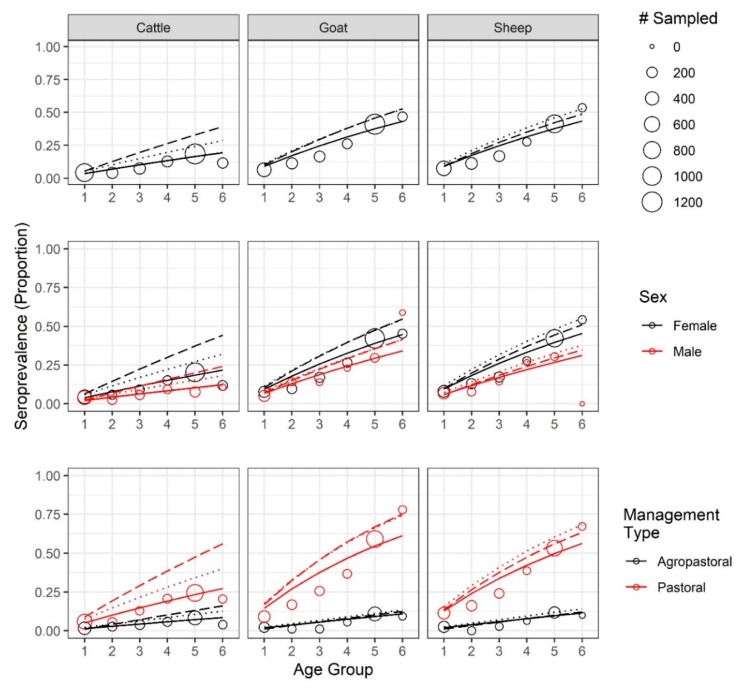
PPRV seroprevalence increases by age for sheep, goats, and cattle. Age–seroprevalence curves by species, sex, and management system. Solid lines indicate the catalytic model fit to the apparent seroprevalence. True seroprevalence adjusted [28] for competitive ELISA antibody test sensitivity and specificity estimates of Couacy-Hymann et al. 2007 [24] and estimates generated in-house (see Methods) are plotted as dotted and dashed lines, respectively.

Age–seroprevalence curves, for both apparent and true seroprevalence (adjusted for cELISA test specificity and sensitivity, see Methods), are presented in Figure 3. Seroprevalence rose with age. In the oldest age cohort, the observed seroprevalence for sheep, goats, and cattle reached 53.6%, 46.8%, and 11.6%, respectively, while the highest adjusted, true seroprevalence reached 52.7%, 52.8%, and 39.2%, respectively. Both the apparent and true sheep and goat seroprevalence were significantly different from the cattle seroprevalence (*p* < 0.001). Notably, after adjustment, the true cattle seroprevalence was 3.4 times the apparent cattle seroprevalence, reflecting the low sensitivity of the cELISA test in cattle. 

For most age groups (Figure 3, Table S3), the females of any species had a higher apparent seroprevalence than males, except for male goats in the second and last age group; however, the only significant sex differences in apparent seroprevalence were found among goats (*p* < 0.02) and cattle (*p* < 0.001) in age group 5. There were not enough male sheep (*n* = 1) in the oldest age group to make comparisons, but the oldest male goats and cattle were significantly different from each other, as were the oldest female sheep and goats when compared with the cattle (*p* <0.001) but not with each other (*p* = 0.26). When adjusted, females had higher true seroprevalence than males across all ages.

Pastoral animals had higher apparent and true seroprevalence in each age group (Figure 3, Table S4), with the oldest animals reaching an apparent seroprevalence of 67.2%, 77.9%, and 20.5%, and highest true seroprevalence of 68.2%, 75.3%, and 56% in sheep, goats, and cattle, respectively. The oldest agropastoral animals reached an apparent seroprevalence of 10.0%, 9.4%, and 4.0%, and highest true seroprevalence of 14.0%, 13.5%, 16.1% in sheep, goats, and cattle, respectively. In pastoral systems, the oldest sheep and goats were significantly different from cattle (*p* << 0.001) but not from each other (*p* = 0.22). In agropastoral systems, there was no significant difference between any pair of species (*p* > 0.28). Within each species, apparent seroprevalence was significantly different between management systems for each age group (*p* <0.05), with the exception of cattle in age group 2 (*p* = 0.44). After adjustment, all comparisons were significantly different. Strikingly, the oldest cattle seroprevalence estimate tripled in pastoral systems and quadrupled in agropastoral systems.

**Figure 4:**
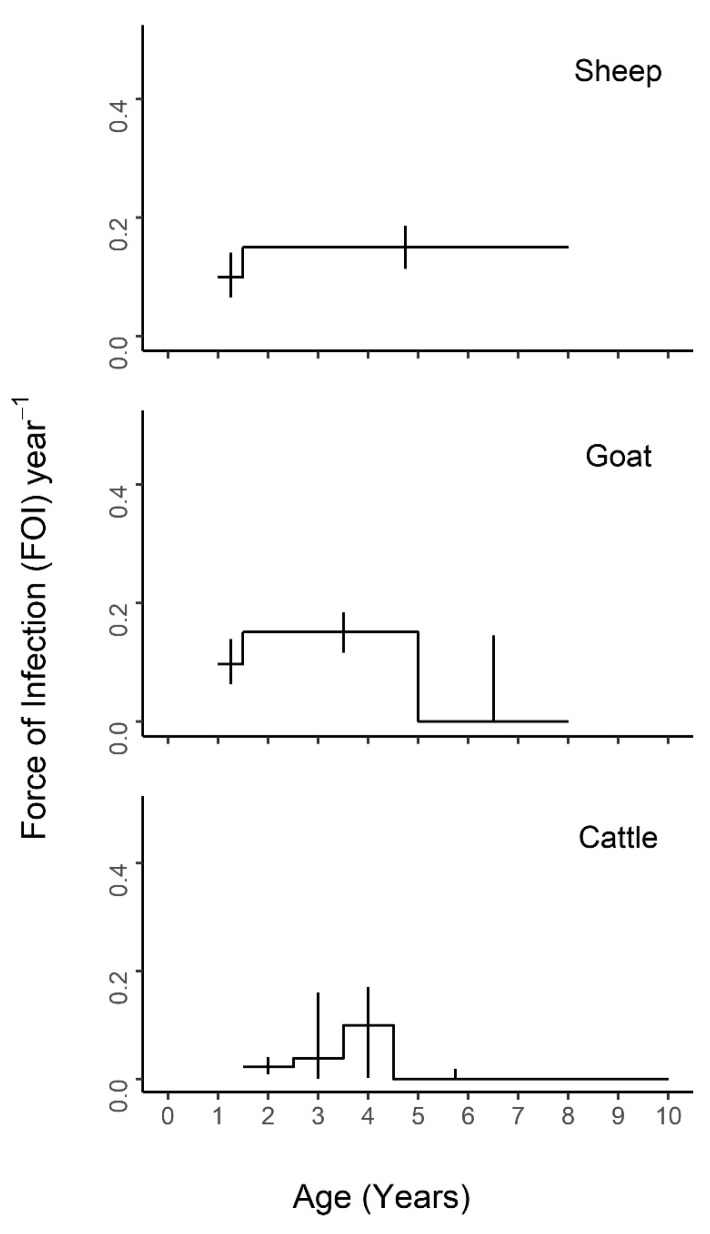
Age-varying force of infection models are a better fit than constant models for PPRV transmission by species. Age-specific force of infection estimates and profile confidence intervals from the best fit models for each species from a piece-wise catalytic model.

**Figure 5:**
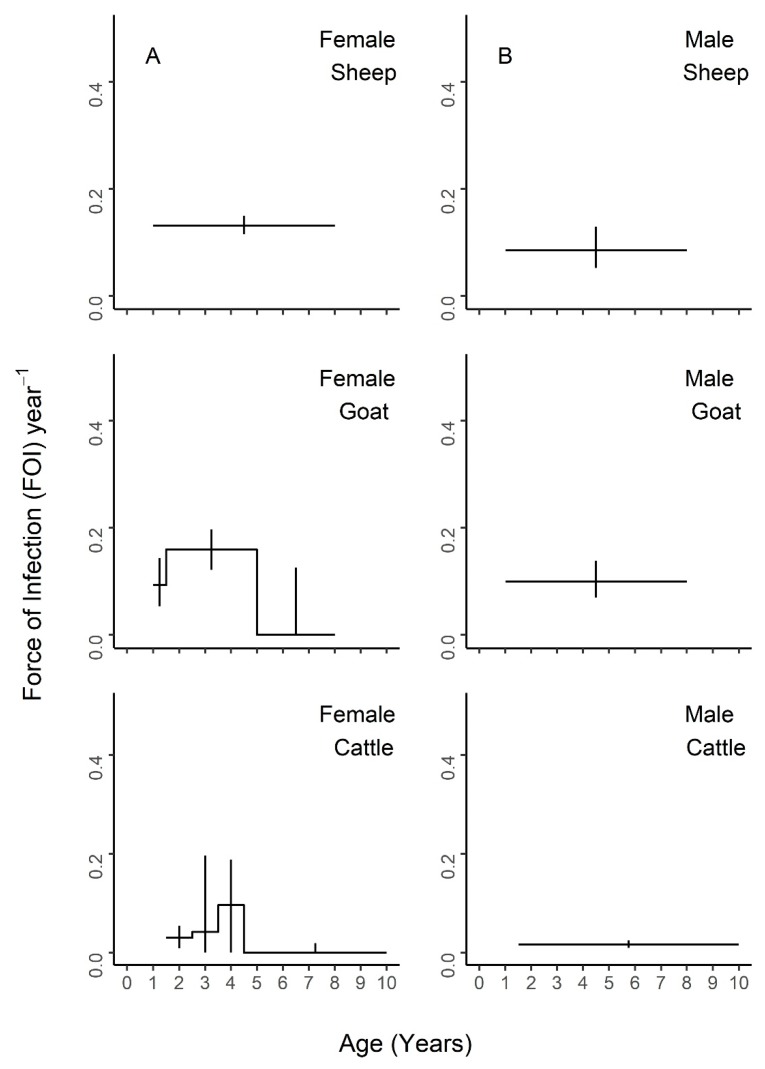
Age-varying force of infection models are a better fit for female goats and cattle than constant models for PPRV transmission by species and sex. Age-specific force of infection estimates and profile confidence intervals from the best fit models for each species from a piece-wise catalytic model, stratified by sex. (**A**) Females (**B**) Males.

**Figure 6:**
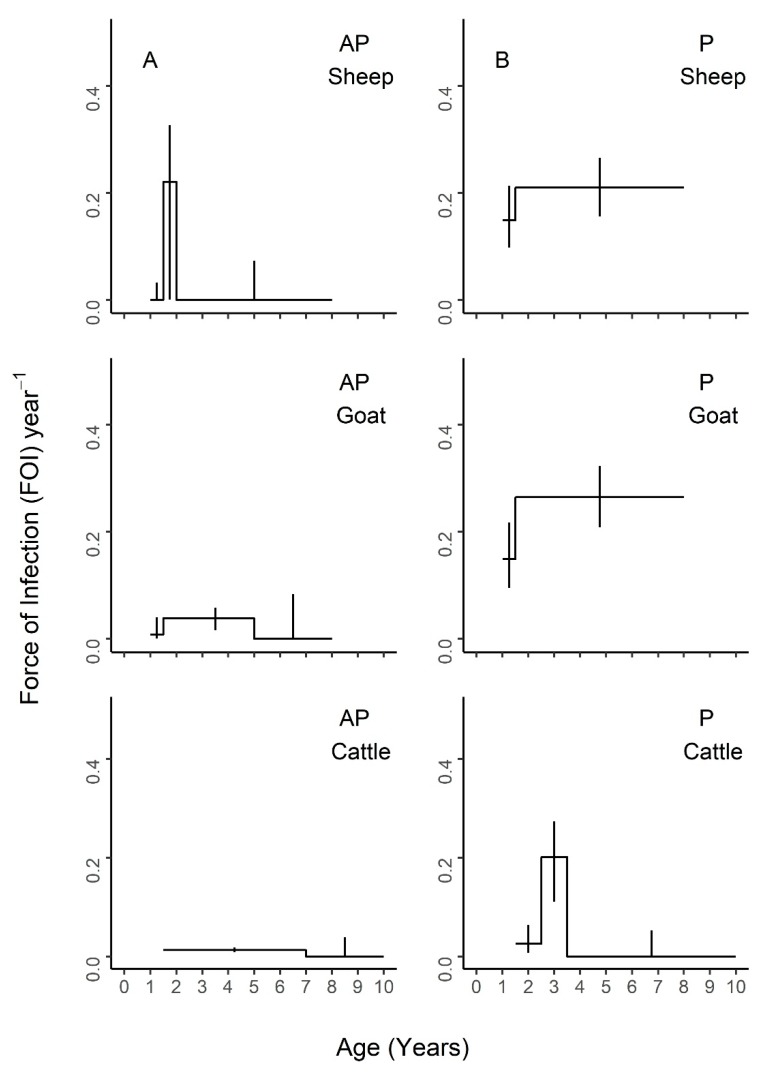
Age-varying force of infection models are a better fit than constant models for PPRV transmission by species and management system. Age-specific force of infection estimates and profile confidence intervals from the best fit models for each species from a piece-wise catalytic model, stratified by management system. (**A**) Agropastoral (AP) (**B**) Pastoral (P)**.**

Nested models comprised of different combinations of neighboring age intervals were compared to the maximal model of all five age groups (Figure S2) and the constant model of one FOI estimate across all ages (Tables S8–S10). The best fit models for each species are presented in Figure 4, Figure 5 and Figure 6, with age-specific FOI estimates represented as a step function. For sheep, the best fit model had two age groups of 1–1.5 and 1.5–8 years, with the second age group having the highest FOI. For goats, the best fit model had three groups of 1–1.5, 1.5–5, and 5–8 years, with the middle age group having the highest FOI and the first age group having the second highest. For cattle, the best fit model included four age groups of 1.5–2.5, 2.5–3.5, 3.5–4.5, and 4.5–10 years, with a clear FOI peak in the 3.5–4.5 age group, followed by the 2.5–3.5 age group. Profile confidence intervals (see Methods) around these best fitting models overlapped, indicating that the FOI estimates for each age group were not significantly different. When stratified by sex (Figure 5), the best fit models were constant for males of all species, as well as female sheep, but were variable for female goats (three age groups) and cattle (four age groups). However, the confidence intervals for the sex- and age-specific FOI estimates overlapped within each of these models. When stratified by management system (Figure 6), the best fit models had varying numbers of age groups, all with overlapping FOI estimates, with the exception of pastoral cattle, which had a significantly different FOI estimate in the 2.3–3.5 year age group when compared to the other two age groups in the best fit model. 

Lastly, logistic regression revealed that the impact of management system on PPRV seroconversion, as measured by the risk ratio, was higher than all but the oldest age group (Figure S3), which had a comparable impact when the confidence intervals were considered. The risk ratio for each species increased with age group as cumulative exposure increased over time.

## 4. Discussion 

Our study has demonstrated that (i) PPRV seroprevalence increased across age for all species, which is consistent with a pattern of endemic infection in which individuals’ cumulative exposure increases with age, (ii) an age-varying model with a variable number of age groups by species provided a better fit to the data, although age-specific FOI estimates were not significantly different from each other in most models, and (iii) FOI estimates for all species were highest in younger age groups. PPRV seroprevalence was significantly higher in sheep and goats than in cattle. Notably, the adjusted true cattle seroprevalence was triple the apparent seroprevalence (quadrupled in agropastoral systems and tripled in pastoral systems). In the case of pastoral cattle, the adjustment resulted in a true seroprevalence of 56%, which is greater than the highest reported cattle seroprevalence estimates previously reported in the literature: 41.9% and 42% [32,33]. This result supports our previous finding [19] that cattle may play a more important role in PPRV transmission than previously realized and also supports the importance of conducting more PPRV research in other species, as called for by members of the PPR Global Research and Expertise Network (GREN) [4]. Across all ages, true PPRV seroprevalence was higher in females than in males and higher in pastoral management systems than in agropastoral systems. This was also true for the FOI for most age groups. The specific age patterns observed suggest a range of biological mechanisms for future study, including the relationship between PPRV seroconversion and the age at first kidding, age at first market debut or mixing outside the herd, age at weaning, and age at waning of maternal immunity across production systems. Although the best fit models had an age-varying FOI, the FOI estimates within most of these models were not significantly different due to the overlapping confidence interval estimates. This suggests that age may not play a strong role in PPRV transmission in the rural Tanzanian setting, with the exception of the significant signal observed in 2.5–3.5-year-old pastoral cattle. Cattle and pastoral systems must be investigated in more detail to explain this finding. Taken together, the data presented here do not support targeted control by age group. Instead, they suggest that targeted control based on other risk factors, such as management system type, may be more effective [19].

These data suggest that all species experience higher FOI estimates when entering their peak reproductive ages. Breeding in Tanzania tends to be uncontrolled, and the biological milestones of age at first breeding or kidding for sheep and goats have been estimated to range from 13.6 to 16.8 months [34,35] and from 44.6 to 48 months for cattle [36,37], with average kidding intervals of 8–12 months for sheep and goats [34,38] and 12–26 months for cattle [36,37]. These estimates align well with the FOI peaks seen in our maximal (Figures S1-S2) and best fit models (Figure 4, Figure 5 and Figure 6). Outside of Tanzania, Dereje et al. [39] reviewed several indigenous breeds in Ethiopia and across Africa and found the age at first kidding to range between 9–24 months, with a kidding interval between 7.9–12 months for goats depending on breed, location, and management system, with the earlier age at first kidding occurring more often in extensive, traditional management systems. Interestingly, the best fit models by sex are constant for males but variable for female goats and cattle, suggesting potential subtle variation in PPRV transmission risk due to sex. This may possibly be due to within-host factors, such as increased female susceptibility to infection due to immune changes during pregnancy (i.e., a periparturient response). However, it seems most likely that non-reproductive behavioral and management factors that lead to increased mixing at reproductive ages may drive the majority of observed age and sex PPRV transmission patterns. Increased mixing could increase transmission through the following mechanisms: i) interactions with animals at the markets where they are taken for sale, and sometimes return, at specific ages or maximum reproductive potential (2–4 years in Tanzanian settings, S. Cleaveland personal communication 2019), or ii) limited mixing of the youngest animals with older animals as part of husbandry practices (e.g., grazing or confinement overnight), or iii) husbandry mating system practices (e.g., increased temporary interaction with breeding male animals for servicing the herd), or iv) increased interaction with young animals who, after losing maternal immunity and prior to vaccination, become infected and pass PPRV to their mothers or other milking females they access prior to weaning between 3 and 6 months [40,41] (aligning with the higher seroprevalence and FOI estimates seen in females in this study). 

Another important age-specific signal considered was maternal immunity. Maternal immunity to PPRV declines dramatically after birth, falling below the protective level by three [13,17], four [15], or five [14] months of age (note: Although these studies cited a protective level, there is not consensus on the level of antibodies necessary to provide protection, nor if antibodies alone are responsible for protection). Animals in this study were at least six months of age, so maternal immunity was not expected to play a role in the FOI patterns presented. Nevertheless, a six age group model (Figure S1, Tables S5–S7) and five age group model (Figure S2, Tables S8–S10) were explored, but neither were selected as the best fitting model among the nested models. FOI differences between these two models may be due to (i) age misclassification among the youngest age group (i.e., they were younger than assessed) or (ii) extended maternal immunity beyond the average weaning age (sheep and goats: 5 months [38], cattle: 7 months [36]) due to prolonged weaning or a differential length of maternal immunity provided by naturally infected mothers (expected in our sample) versus vaccinated mothers [13,14,42]. Future experiments are needed to clarify the role of immunity type and breed on maternal immunity duration as well as to examine PPRV transmissibility to young via milk [43] or reproductive tract secretions. Although our data do not support targeted control by age group, determining the duration of maternal immunity could allow for the effective, targeted vaccination of young susceptibles, which may be cost effective in endemic settings.

The finding that the apparent and true age–seroprevalence curves did not rise above 60% in the oldest age group when stratified by species or by species and sex is unexpected for a fully immunizing infection in an endemic setting. When stratified by management system, pastoral sheep and goats’ age–seroprevalence curves rose to the high proportion expected in endemic settings, whereas the curves among agropastoral animals did not, suggesting that PPRV may not be endemic in agropastoral production systems (in agreement with PPRV modelling studies in Ethiopia [44]) and that management system differences explain the unexpected, slow rise observed when stratifying only by species, or by species and sex. Age–seroprevalence curves may also be affected by infection-associated mortality in sheep and goats, which would cause left truncation in our cross-sectional dataset and underestimation of the FOI (possibly more pronounced the smaller the FOI [45]). All animals sampled in this study were clinically healthy, and no case fatality data were available to correct for infection-associated mortality among sheep and goats. Cattle are not known to suffer mortality from PPRV (although clinical disease and mortality have been seen among Asian water buffalo [46], a non-primary host), so this effect is not expected to impact cattle age–seroprevalence curves, though further investigations are likely needed here. Currently, the available herd-level case fatality data in endemic settings are limited, and the variance is wide, which has been attributed to factors such as strain, species, and breed [18]. Where possible, future studies should strive to collect detailed case fatality data from recent or current epidemics in the study area to enable improved seroprevalence and FOI estimates in future cross-sectional studies.

Our study made several assumptions including the following: PPRV endemicity in Tanzania; a positive cELISA result indicates past PPRV exposure and current protection; a negative cELISA result indicates no past exposure and current susceptibility; that the BDSL cELISA kit was suitable for testing cattle samples; and that cross-reactivity with rinderpest or rinderpest-like viruses [47] was not expected in our samples given the sampling date and methods used to develop the cELISA kit. These assumptions are the same as those for the catalytic model with the assumption of constant age and have been discussed as reasonable first approximations for understanding PPRV transmission dynamics in previous work [19]. Additionally, while the analytical approach taken in this study can identify which age cohorts are responsible for the majority of transmission, it cannot distinguish whether PPRV infection occurs mainly within a given age class or whether it originates from a different age class [29]. Age misclassification in this study may have increased variation in FOI estimates in the last age group by classifying more animals in the second to last age group (full mouth) than in the last age group (full mouth plus wear) due to the difficulty of assessing age by variation in dental wear in full-mouthed animals. Lastly, we previously discussed the role of cattle in PPRV transmission in [19]. Two recent experimental transmission trials involving cattle did not find onward transmission from cattle [48,49]. This is an active area of research, as variation due to the use of local vs. non-local breeds and the choice of PPRV isolate may impact the results. It will be important to determine if cattle or other atypical hosts [50] can transmit PPRV onward as part of the upcoming PPRV global eradication campaign.

This study has demonstrated that the force of infection of PPRV varies significantly by age for pastoral cattle and that non-significant but age-varying force of infection patterns are present among sheep, goats, and cattle. Management practices and biological milestones that occur near the identified force of infection peak ages should be targeted for further study. These data do not support targeted control by age group. Instead, they suggest that targeted control based on management systems may be more effective to achieve PPRV eradication. 

## Data Availability

The anonymized dataset used in this study will be made available on the following repository: http://researchdata.gla.ac.uk/

## Supplementary Materials

The following are available online at https://www.mdpi.com/1999-4915/12/2/186/s1, Figure S1: Age-specific force of infection estimates from a piece-wise catalytic model with six age groups and age-seroprevalence curves by species, Figure S2: Age-specific force of infection estimates from a piece-wise catalytic model with five age groups and age-seroprevalence curves by species; Figure S3: Logistic regression estimates of the impact of age, management, and sex on PPRV seroconversion; Table S1: Sample population characteristics, modified from Herzog et al 2019; Table S2: Sample population distribution and apparent PPRV seroprevalence by dentition-based age group; Table S3: Sample population distribution and apparent PPRV seroprevalence by dentition-based age group and sex; Table S4: Sample population distribution and apparent PPRV seroprevalence by dentition-based age group and management system; Table S5: Sheep nested models and AIC values for models combining six age groups; Table S6: Goat nested models and AIC values for models combining six age groups; Table S7: Cattle nested models and AIC values for models combining six age groups; Table S8: Sheep nested models and AIC values for models combining five age groups; Table S9: Goat nested models and AIC values for models combining five age groups; Table S10: Cattle nested models and AIC values for models combining five age groups; Text S1: Additional References that Tested the Significance of Age for PPRV Seroprevalence.
