# Supplementary material for: Identifying Age Cohorts Responsible for Peste Des Petits Ruminants Virus Transmission among Sheep, Goats, and Cattle in Northern Tanzania
Source: Viruses. 2020 Feb 7;12(2):186. doi: 10.3390/v12020186 (PMC7077219; doi:10.3390/v12020186)
Supplement: Supplementary file 1 [file viruses-12-00186-s001.pdf]

Table S1. Sample population characteristics, modified from Herzog et al 2019 [1]

	n (%)	PPRV Seropositive n (%, *95% CI*)	Adjusted Seroprevalence†† (%, 95% CI)
Total	7,496	1,580 (21.1%, *20.2-22.0%*)	
Species			
Sheep	2,080 (27.7%)	545 (26.2%, *24.3-28.1%*)	29.0%
Goats	2,419 (32.3%)	696 (28.8%, *27.0-30.6%*)	34.8%
Cattle	2,997 (40%)	339 (11.3%, *10.2-12.5%*)	22%
Sex			
Female	5,508 (73.5%)	1,393 (25.3%, *24.1-26.4%*)	
Male	1,988 (26.5%)	187 (9.4%)	
Management system			
Agropastoral (AP)	2,898 (38.7%)	169 (5.8%, *5.0-6.7%*)	
Pastoral (P)	4,598 (61.3%)	1,411 (30.7%, *29.4-32.0%*)	
Age Group†			
Temporary teeth	1,869 (24.9%)	106 (5.7%, *4.7-6.8%*)	
1 pair	757 (10.1%)	68 (9.0%, *7.0-11.2%*)	
2 pair	666 (8.9%)	88 (13.2%, *10.7-16.0%*)	
3 pair	483 (6.4%)	97 (20.1%, *16.6-23.9%*)	
4 pair	3,307 (44.1%)	1,088 (32.9%, 31.3-34.5%)	
Full mouth + worn	414 (5.5%)	133 (32.1%, *27.6-36.9%*)	

† Age groups by dentition correspond to approximately the following ages in cattle: temporary teeth: 1 month to 1.5 years; 1 pair: 1.5-2.5 years; 2 pair: 2.5-3.5 years, 3 pair: 3.5-4.5 years, 4 pair: 4.5-7 years; full + worn: above 7 years. For sheep and goats: temporary teeth: < 1 year; 1 pair: 1-1.5 years; 2 pair: 1.5-2 years, 3 pair: 2-3 years, 4 pair: 3-5 years; full + worn: above 5 years.

†† Species specific sensitivity and specificity estimates from Logan et al 2019 (unpublished). Adjustment according to Rogan & Gladen 1978 [2].

CI : Confidence Interval

Table S2. Sample population distribution and apparent PPRV seroprevalence by dentition-based age group

	Age Group n (%)						Age Group PPRV Seropositive n (%, 95% CI)					
	**1**	**2**	**3**	**4**	**5**	**6**	**1**	**2**	**3**	**4**	**5**	**6**
**Overall**	1869 (24.9%)	757 (10.1%)	666 (8.9%)	483 (6.4%)	3307 (44.1%)	414 (5.5%)	106 (5.7%, 4.7-6.8%)	68 (9.0%, 7.0-11.2%)	88 (13.2%, 10.7-16.0%)	97 (20.1%, 16.6-23.9%)	1,088 (32.9%, 31.3-34.5%)	133 (32.1%, 27.6-36.9%)
Sheep	524 (25.2%)	277 (13.3%)	209 (10.0%)	94 (4.5%)	892 (42.9%)	84 (4.0%)	39 (7.4%, 5.3-10.0%)	31 (11.2%, 7.7-15.5%)	35 (16.8%, 12.0-22.5%)	26 (27.7%, 18.9-37.8%)	369 (41.4%, 38.1-44.7%)	45 (53.6%, 42.4-64.5%)
Goat	455 (18.8%)	239 (9.9%)	212 (8.7%)	161 (7.6%)	1211 (40.2%)	141 (6.3%)	29 (6.4%, 4.3-9.0%)	27 (11.3%, 7.6-16.0%)	35 (16.5%, 11.8-22.2%)	42 (26.1%, 19.5-33.6%)	497 (41.0%, 38.3-43.9%)	66 (46.8%, 38.4-55.4%)
Cattle	890 (29.7%)	241 (8.0%)	245 (8.2%)	228 (7.6%)	1204 (40.2%)	189 (6.3%)	38 (4.3%, 3.0-5.8%)	10 (4.2%, 2.0-7.5%)	18 (7.4%, 4.4-11.4%)	29 (12.7%, 8.7-17.8%)	222 (18.4%, 16.3-20.7%)	22 (11.6%, 7.4-17.1%)
**Pastoral**	1104 (24.0%)	474 (10.3%)	363 (7.9%)	274 (6.0%)	2154 (46.8%)	229 (5.0%)	90 (8.2%, 6.6-9.9%)	64 (13.5%, 10.6-16.9%)	79 (21.8%, 17.6-26.3%)	85 (31.0%, 25.6-36.9%)	972 (45.1%, 43.0-47.2%)	121 (52.8%, 46.1-59.4%)
**Agro-pastoral**	765 (26.4%)	283 (9.8%)	303 (10.5%)	209 (7.2%)	1153 (39.8%)	185 (6.4%)	16 (2.1%, 1.2-3.4%)	4 (1.4%, 0.4-3.6%)	9 (3.0%, 1.4-5.6%)	12 (5.7%, 3.0-9.8%)	116 (10.1%, 8.4-12.0%)	12 (6.5%, 3.4-11.1%)
**Female**	930 (16.9%)	462 (8.4%)	456 (8.3%)	346 (6.3%)	2946 (53.5%)	368 (6.7%)	59 (6.3%, 4.9-8.1%)	46 (10.0%, 7.4-13.1%)	69 (15.1%, 12.0-18.8%)	78 (22.5%, 18.2-27.3%)	1021 (34.7%, 33.0-36.4%)	120 (32.6%, 27.8-37.7%)
**Male**	939 (47.2%)	295 (14.8%)	210 (10.6%)	137 (6.9%)	361 (18.2%)	46 (2.3%)	47 (5.0%, 3.7-6.6%)	22 (7.5%, 4.7-11.1%)	19 (9.0%, 5.5-13.8%)	19 (13.9%, 8.6-20.8%)	67 (18.6%, 14.7-23.0%)	13 (28.3%, 16.0-43.5%)

Table S3. Sample population distribution and apparent PPRV seroprevalence by dentition-based age group and sex

	Age Group n (%)						Age Group PPRV seropositive n (%, 95% CI)					
	1	2	3	4	5	6	1	2	3	4	5	6
**Overall**	1869 (24.9%)	757 (10.1%)	666 (8.9%)	483 (6.4%)	3307 (44.1%)	414 (5.5%)	106 (5.7%, 4.7-6.8%)	68 (9.0%, 7.0-11.2%)	88 (13.2%, 10.7-16.0%)	97 (20.1%, 16.6-23.9%)	1,088 (32.9%, 31.3-34.5%)	133 (32.1%, 27.6-36.9%)
**Female**	930 (16.9%)	462 (8.4%)	456 (8.3%)	346 (6.3%)	2946 (53.5%)	368 (6.7%)	59 (6.3%, 4.9-8.1%)	46 (10.0%, 7.4-13.1%)	69 (15.1%, 12.0-18.8%)	78 (22.5%, 18.2-27.3%)	1021 (34.7%, 33.0-36.4%)	120 (32.6%, 27.8-37.7%)
Sheep	273 (17.0%)	186 (11.5%)	167 (10.4%)	79 (4.9%)	823 (51.1%)	83 (5.2%)	22 (8.1%, 5.1-11.9%)	24 (12.9%, 8.4-18.6%)	29 (17.4%, 11.9-24.0%)	22 (27.9%, 18.3-39.1%)	348 (42.3%, 38.9-45.7%)	45 (54.2%, 42.9-65.2%)
Goat	222 (11.6%)	155 (8.1%)	169 (8.9%)	135 (7.1%)	1103 (57.8%)	124 (6.5%)	17 (7.7%, 4.5-12.0%)	15 (9.7%, 5.5-15.5%)	29 (17.2%, 11.8-23.7%)	36 (26.7%, 19.4-35.0%)	465 (42.2%, 39.2-45.1%)	56 (45.2%, 36.2-54.3%)
Cattle	435 (21.9%)	121 (6.1%)	120 (6.0%)	132 (6.6%)	1020 (51.3%)	161 (8.1%)	20 (4.6%, 2.8-7.0%)	7 (5.8%, 2.4-11.6%)	11 (9.2%, 4.7-15.8%)	20 (15.2%, 9.5-22.4%)	208 (20.4%, 18.0-23.0%)	19 (11.8%, 7.3-17.8%)
**Male**	939 (47.2%)	295 (14.8%)	210 (10.6%)	137 (6.9%)	361 (18.2%)	46 (2.3%)	47 (5.0%, 3.7-6.6%)	22 (7.5%, 4.7-11.1%)	19 (9.0%, 5.5-13.8%)	19 (13.9%, 8.6-20.8%)	67 (18.6%, 14.7-23.0%)	13 (28.3%, 16.0-43.5%)
Sheep	251 (53.5%)	91 (19.4%)	42 (9.0%)	15 (3.2%)	69 (14.7%)	1 (0.2%)	17 (6.8%, 4.0-10.6%)	7 (7.7%, 3.1-15.2%)	6 (14.3%, 5.4-28.5%)	4 (26.7%, 7.8-55.1%)	21 (30.4%, 19.9-42.7%)	0 (0%, 0-97.5%)
Goat	233 (45.6%)	84 (16.4%)	43 (8.4%)	26 (5.1%)	108 (21.1%)	17 (3.3%)	12 (5.2%, 2.7-8.8%)	12 (14.3%, 7.6-23.6%)	6 (14.0%, 5.3-27.9%)	6 (23.1%, 9.0-43.6%)	32 (29.6%, 21.2-39.2%)	10 (58.8%, 32.9-81.6%)
Cattle	455 (45.1%)	120 (11.9%)	125 (12.4%)	96 (9.5%)	184 (18.3%)	28 (2.8%)	18 (4.0%, 2.4-6.2%)	3 (2.5%, 0.5-7.1%)	7 (5.6% , 2.3-11.2%)	9 (9.4%, 4.4-17.2%)	14 (7.6%, 4.2-12.4%)	3 (10.7%, 2.3-28.2%)

Table S4. Sample population distribution and apparent PPRV seroprevalence by dentition-based age group and management system

	Age Group n (%)						Age Group PPRV seropositive n (%, 95% CI)					
	**1**	**2**	**3**	**4**	**5**	**6**	**1**	**2**	**3**	**4**	**5**	**6**
**Overall**	1869 (24.9%)	757 (10.1%)	666 (8.9%)	483 (6.4%)	3307 (44.1%)	414 (5.5%)	106 (5.7%, 4.7-6.8%)	68 (9.0%, 7.0-11.2%)	88 (13.2%, 10.7-16.0%)	97 (20.1%, 16.6-23.9%)	1,088 (32.9%, 31.3-34.5%)	133 (32.1%, 27.6-36.9%)
**Pastoral**	1104 (24.0%)	474 (10.3%)	363 (7.9%)	274 (6.0%)	2154 (46.8%)	229 (5.0%)	90 (8.2%, 6.6-9.9%)	64 (13.5%, 10.6-16.9%)	79 (21.8%, 17.6-26.3%)	85 (31.0%, 25.6-36.9%)	972 (45.1%, 43.0-47.2%)	121 (52.8%, 46.1-59.4%)
Sheep	290 (21.0%)	192 (13.9%)	137 (9.9%)	62 (4.5%)	637 (46.1%)	64 (4.6%)	33 (11.4%, 8.0-15.6%)	31 (16.2%, 11.2-22.1%)	33 (24.1%, 17.2-32.1%)	24 (38.7%, 26.6-51.9%)	339 (53.2%, 49.3-57.1%)	43 (67.2%, 54.3-78.4%)
Goat	274 (18.2%)	154 (10.3%)	132 (8.8%)	106 (7.1%)	759 (50.5%)	77 (5.1%)	25 (9.1%, 6.0-13.1%)	26 (16.9%, 11.3-23.7%)	34 (25.8%, 18.5-34.1%)	39 (36.8%, 27.6-46.7%)	448 (59.0%, 55.4-62.5%)	60 (77.9%, 67.0-86.6%)
Cattle	540 (31.5%)	128 (7.5%)	94 (5.5%)	106 (6.2%)	758 (44.2%)	88 (5.1%)	32 (5.9%, 4.1-8.3%)	7 (5.5%, 2.2-10.9%)	12 (12.8%, 6.8-21.2%)	22 (20.8%, 13.5-29.7%)	185 (24.4%, 21.4-27.6%)	18 (20.5%, 12.6-30.4%)
**Agro-pastoral**	765 (26.4%)	283 (9.8%)	303 (10.5%)	209 (7.2%)	1153 (39.8%)	185 (6.4%)	16 (2.1%, 1.2-3.4%)	4 (1.4%, 0.4-3.6%)	9 (3.0%, 1.4-5.6%)	12 (5.7%, 3.0-9.8%)	116 (10.1%, 8.4-12.0%)	12 (6.5%, 3.4-11.1%)
Sheep	234 (33.5%)	85 (12.2%)	72 (10.3%)	32 (4.6%)	255 (36.5%)	20 (2.9%)	6 (2.6%, 0.9- 5.5%)	0 (0%, 0-4.2%)	2 (2.8%, 0.3-9.7%)	2 (6.3%, 0.8-20.8%)	30 (11.8%, 8.1-16.4%)	2 (10.0%, 1.2-31.7%)
Goat	181 (19.7%)	85 (9.3%)	80 (8.7%)	55 (6.0%)	452 (49.3%)	64 (7.0%)	4 (2.2%, 0.6-5.6%)	1 (1.2%, 0.03-6.4%)	1 (1.3%, 0.03-6.8%)	3 (5.5%, 1.1-15.1%)	49 (10.8%, 8.1-14.1%)	6 (9.4%, 3.5-19.3%)
Cattle	350 (27.3%)	113 (8.8%)	151 (11.8%)	122 (9.5%)	446 (34.8%)	101 (7.9%)	6 (1.7%, 0.6-3.7%)	3 (2.7%, 0.6-7.6%)	6 (4.0%, 1.5-8.4%)	7 (5.7%, 2.3-11.5%)	37 (8.3%, 5.9-11.3%)	4 (4.0%, 1.1-9.8%)

Table S5. Sheep nested models and AIC values for models combining six age groups

Model Age Intervals	Number of Intervals	AIC Values
0-1.5, 1.5-2, 2-3, 3-5, 5-8	5	47.11
0-1, 1-1.5, 1.5-2, 2-3, 3-5, 5-8 (maximal model)	6	45.9
0-2, 2-3, 3-5, 5-8	4	45.7
0-1, 1-2, 2-3, 3-5, 5-8	5	45.38
0-1, 1-3, 3-5, 5-8	4	45.07
0-1, 1-5, 5-8	3	44.69
0-1, 1-1.5, 1.5-2, 2-5, 5-8	5	44.01
0-1, 1-1.5, 1.5-2, 2-3, 3-8	5	44
0-1, 1-1.5, 1.5-3, 3-5, 5-8	5	43.9
0-3, 3-5, 5-8	3	43.62
0-1, 1-8	2	43.19
0-5, 5-8	2	43.16
0-1, 1-1.5, 1.5-5, 5-8	4	42.14
0-1, 1-1.5, 1.5-2, 2-8	4	42.75
0-1, 1-1.5, 1.5-8	3	41.61
**0-8 (constant model)**	**1**	**41.47**

Table S6. Goat nested models and AIC values for models combining six age groups

Model Age Intervals	Number of Intervals	AIC Values

0-1, 1-1.5, 1.5-8	3	231.23
0-1, 1-1.5, 1.5-2, 2-8	4	46.93
0-1, 1-1.5, 1.5-2, 2-3, 3-8	5	46.58
0-1, 1-8	2	46.44
0-1, 1-1.5, 1.5-2, 2-3, 3-5, 5-8 (maximal model)	6	45.85
0-1.5, 1.5-2, 2-3, 3-5, 5-8	5	45.04
0-8 (constant model)	1	44.93
0-1, 1-2, 2-3, 3-5, 5-8	5	44.7
0-1, 1-3, 3-5, 5-8	4	44.24
0-1, 1-1.5, 1.5-2, 2-5, 5-8	5	44
0-1, 1-1.5, 1.5-3, 3-5, 5-8	5	43.91
0-2, 2-3, 3-5, 5-8	4	43.22
0-1, 1-5, 5-8	3	42.67
0-5, 5-8	2	42.43
0-1, 1-1.5, 1.5-5, 5-8	4	42.38
**0-3, 3-5, 5-8**	**3**	**42.29**

Table S7. Cattle nested models and AIC values for models combining six age groups

Model Age Intervals	Number of Intervals	AIC Values
0-1.5, 1.5-10	2	103.45

0-10 (constant model)	1	67.66
0-1.5, 1.5-2.5, 2.5-10	3	63.32
0-7, 7-10	2	60.72
0-1.5, 1.5-4.5, 4.5-7, 7-10	4	60.52
0-3.5, 3.5-4.5, 4.5-7, 7-10	4	58.85
0-4.5, 4.5-7, 7-10	3	58.78
0-1.5, 1.5-2.5, 2.5-3.5, 3.5-10	4	58.22
0-1.5, 1.5-7, 7-10	3	58.09
0-2.5, 2.5-3.5, 3.5-4.5, 4.5-7, 7-10	5	58.06
0-1.5, 1.5-2.5, 2.5-3.5, 3.5-7, 7-10	5	57.52
0-1.5, 1.5-2.5, 2.5-7, 7-10	4	56.78
0-1.5, 1.5-2.5, 2.5-4.5, 4.5-7, 7-10	5	53.69
0-1.5, 1.5-2.5, 2.5-3.5, 3.5-4.5, 4.5-7, 7-10 (maximal model)	6	53.54
0-1.5, 1.5-3.5, 3.5-4.5, 4.5-7, 7-10	5	51.62
**0-1.5, 1.5-2.5, 2.5-3.5, 3.5-4.5, 4.5-10**	**5**	**51.54**

Table S8. Sheep nested models and AIC values for models combining five age groups

Model Age Intervals	Number of Intervals	AIC Values
1-1.5, 1.5-2, 2-3, 3-5, 5-8 (maximal model)	5	37.25
1-5, 5-8	2	36.41
1-1.5, 1.5-2, 2-5, 5-8	4	35.5
1-1.5, 1.5-2, 2-3, 3-8	4	35.47
1-2, 2-3, 3-5, 5-8	4	35.43

1-1.5, 1.5-3, 3-5, 5-8	4	35.28
1-8 (constant model)	1	34.63
1-3, 3-5, 5-8	3	34.62
1-1.5, 1.5-2, 2-8	3	34.17
1-1.5, 1.5-5, 5-8	3	33.49
**1-1.5, 1.5-8**	**2**	**32.74**

Table S9. Goat nested models and AIC values for models combining five age groups

**Model Age Intervals**	**Number of Intervals**	**AIC Values**
1-1.5, 1.5-8	2	40.1
1-1.5, 1.5-2, 2-8	3	40.01
1-8 (constant model)	1	39.66
1-1.5, 1.5-2, 2-3, 3-5, 5-8 (maximal model)	5	38.57
1-5, 5-8	2	37.24
1-1.5, 1.5-2, 2-3, 3-8	4	37.23
1-1.5, 1.5-2, 2-5, 5-8	4	37.16
1-1.5, 1.5-3, 3-5, 5-8	4	36.63
1-2, 2-3, 3-5, 5-8	4	36.59
1-3, 3-5, 5-8	3	36.48
**1-1.5, 1.5-5, 5-8**	**3**	**34.79**

Table S10. Cattle nested models and AIC values for models combining five age groups

**Model Age Intervals**	**Number of Intervals**	**AIC Values**
1.5-2.5, 2.5-10	2	52.52
1.5-10 (constant model)	1	50.52
1.5-4.5, 4.5-7, 7-10	3	48.42

1.5-2.5, 2.5-3.5, 3.5-7, 7-10	4	46.33
1.5-2.5, 2.5-7, 7-10	3	45.39
1.5-7, 7-10	2	44.48
1.5-2.5, 2.5-3.5, 3.5-10	3	44.31
1.5-2.5, 2.5-3.5, 3.5-4.5, 4.5-7, 7-10 (maximal model)	5	41.5
1.5-2.5, 2.5-4.5, 4.5-7, 7-10	4	40.17
1.5-3.5, 3.5-4.5, 4.5-7, 7-10	4	39.63
**1.5-2.5, 2.5-3.5, 3.5-4.5, 4.5-10**	**4**	**39.5**

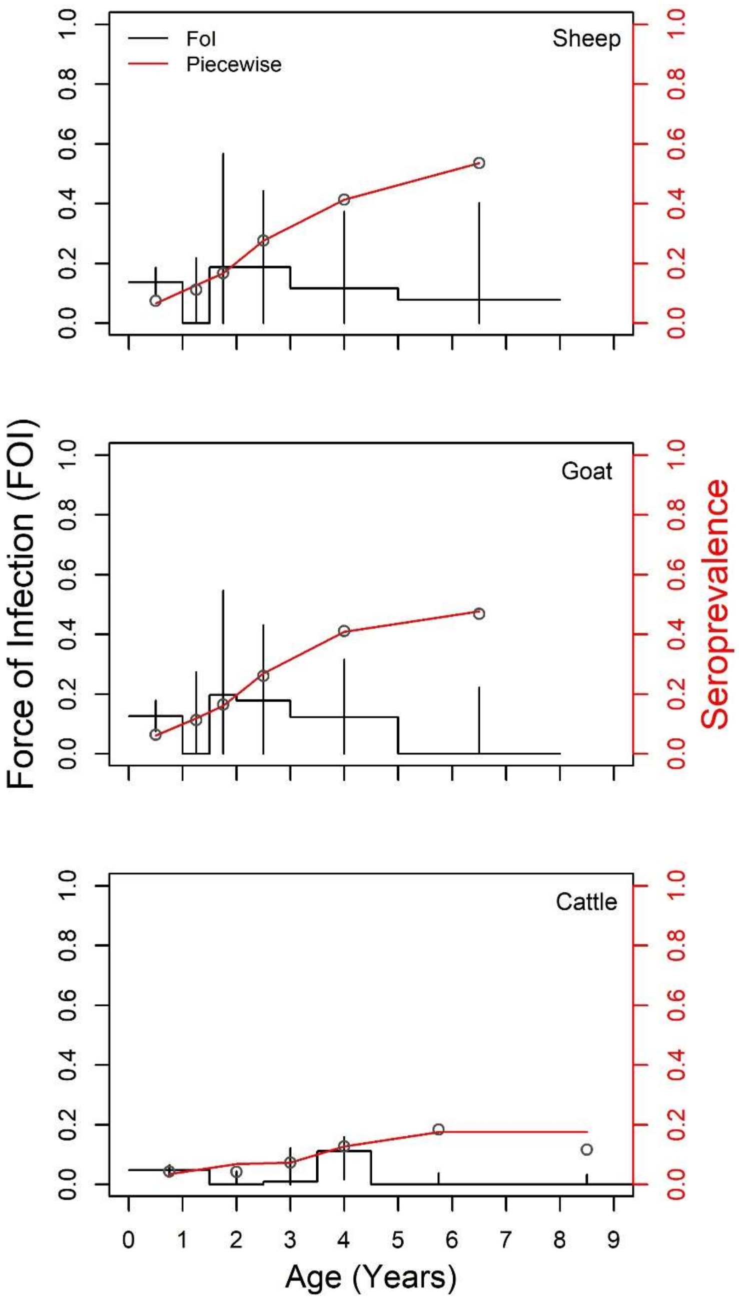

Figure S1. Age-specific force of infection estimates from a piece-wise catalytic model with six age groups and age-seroprevalence curves by species. The model fit is plotted as a line, age group seroprevalence estimates as points, and the age-specific FOI estimates as a step function.

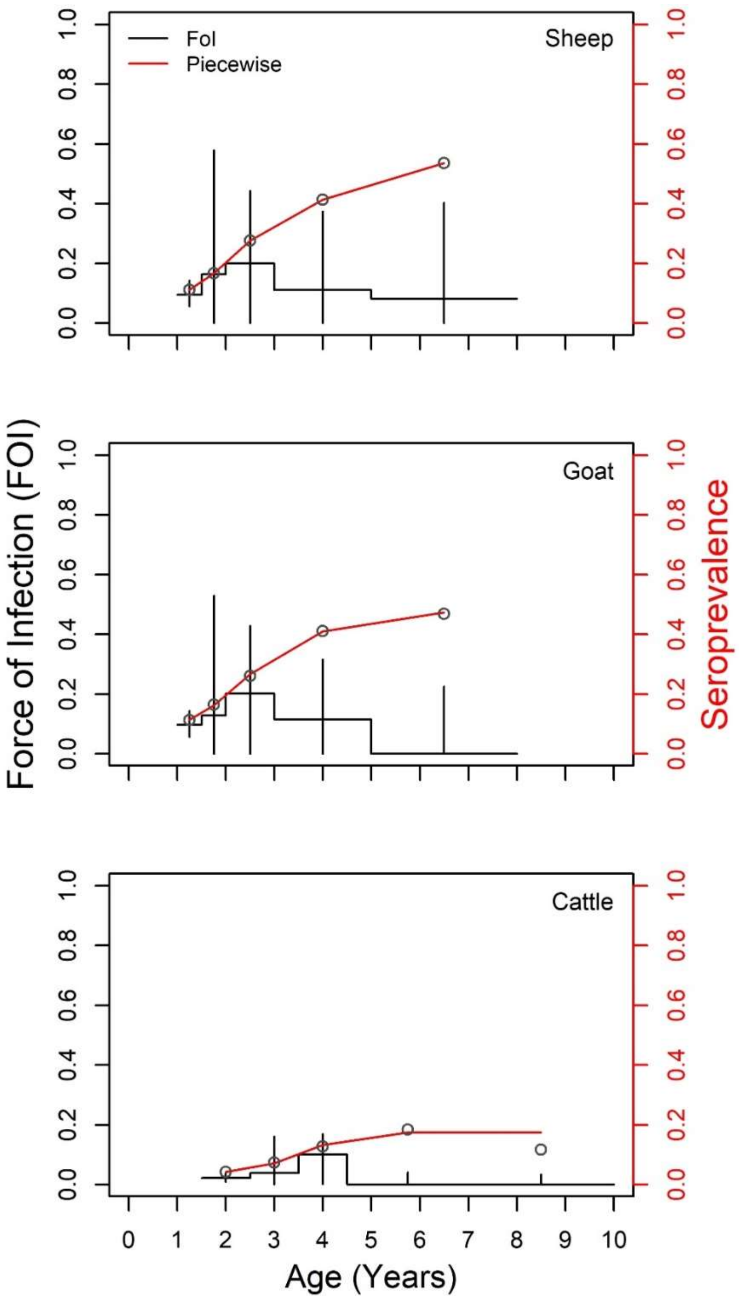

Figure S2. Age-specific force of infection estimates from a piece-wise catalytic model with five age groups and age-seroprevalence curves by species. The model fit is plotted as a line, age group seroprevalence estimates as points, and the age-specific FOI estimates as a step function.

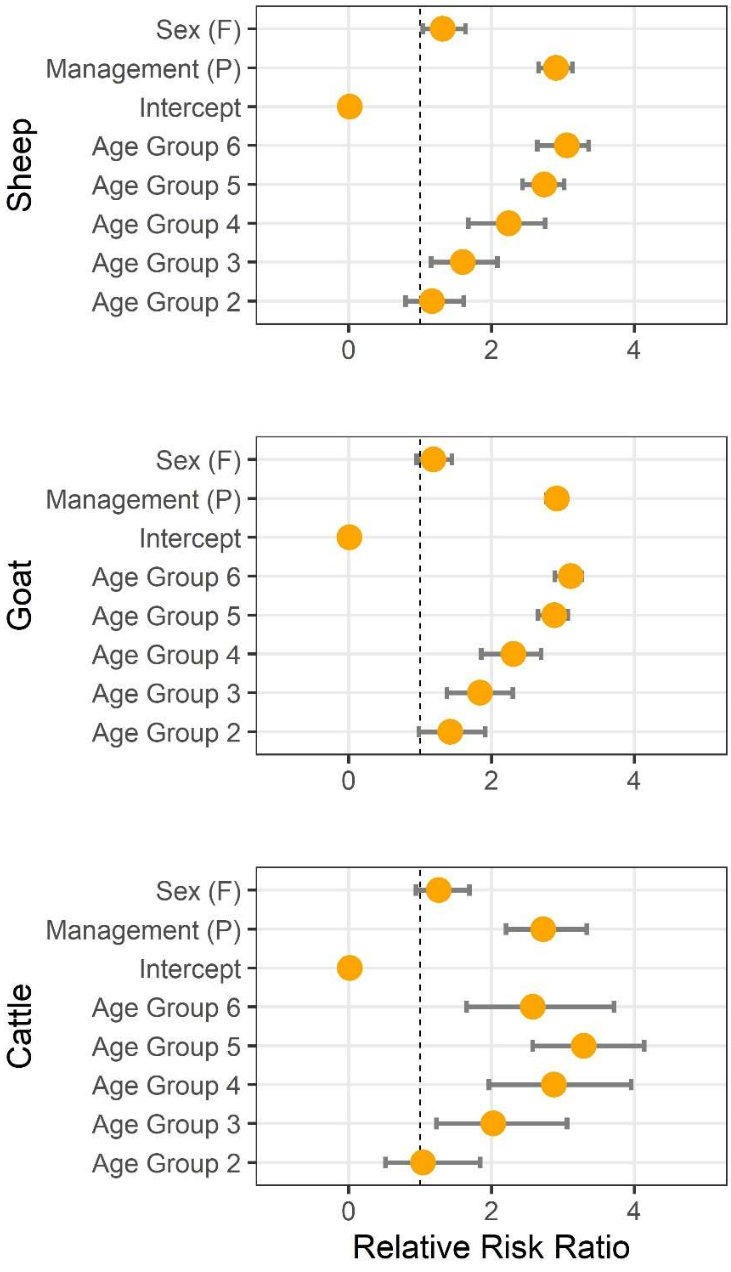

Figure S3. Logistic regression estimates of the impact of age, management, and sex on PPRV seroconversion. Reference group: male agropastoral cattle in age group 1. Management has a greater risk ratio and greater impact than all but the oldest age group(s).

Supplemental Text. Additional References that Tested the Significance of Age for PPRV Seroprevalence

Significant Age

The significance of age is supported by twenty-one studies that investigated two [3–8], three [9,10,19,20,11–18], four (Dejene 2016, unpublished master thesis) [21,22], or five [23] age groups and also found PPRV seroprevalence to increase with age. Four studies reported a significant effect of age, but did not find that PPRV seroprevalence increased with age [8,24–26] as would be expected with a fully immunizing infection.

Non-significant Age

The thirteen studies that found no significant effect of age had an unknown amount of age groups [27], two age groups [28–37], or three age groups [38,39].

References

1. **Herzog CM, *et al.*** Pastoral production is associated with increased peste des petits ruminants seroprevalence in northern Tanzania across sheep, goats and cattle. *Epidemiology and Infection* 2019; **147**: 1–9.
2. **Rogan WJ, Gladen B**. Estimating Prevalence from the Results of a Screening Test. *American Journal of Epidemiology* 1978; **107**: 71–76.
3. **Kivaria FM, *et al.*** The incursion, persistence and spread of peste des petits ruminants in Tanzania: Epidemiological patterns and predictions. *The Onderstepoort Journal of Veterinary Research* 2013; **80**: 1–10.
4. **Kardjadj M, *et al.*** Seroprevalence, distribution and risk factor for peste des petits

ruminants (PPR) in Algeria. *Preventive Veterinary Medicine* 2015; **122**: 205–210.

5. **Al-Majali AM, *et al.*** Seroprevalence of, and risk factors for, peste des petits ruminants in sheep and goats in northern Jordan. *Preventive Veterinary Medicine* 2008; **85**: 1–8.
6. **Gari G, *et al.*** Serological investigation of peste des petits ruminants in east Shewa and Arsi zones, Oromia region, Ethiopia. *Veterinary Medicine International* 2017; **2017**: 1–5.
7. **Kulkarni D, *et al.*** Peste des petits ruminants in goats in India. *The Veterinary Record* 1996; **138**: 187–188.
8. **Elhaig MM, *et al.*** Prevalence and molecular characterization of peste des petits ruminants virus from Ismailia and Suez, Northeastern Egypt, 2014–2016. *Small Ruminant Research* 2018; **169**: 94–98.
9. **Torsson E, *et al.*** Seroprevalence and risk factors for peste des petits ruminants and selected differential diagnosis in sheep and goats in Tanzania. *Infection Ecology & Epidemiology* Taylor & Francis, 2017; **7**: 1368336.
10. **Salih HAM, *et al.*** Seroprevalence and risk factors of peste des petits ruminants in sheep and goats in Sudan. *Journal of Advanced Veterinary and Animal Research* 2014; **1**: 42–49.
11. **Mahajan S, *et al.*** Risk of seroconversion to peste des petits ruminants (PPR) and its association with species, sex, age and migration. *Small Ruminant Research* 2012; **104**: 195–200.
12. **Patel YR, *et al.*** Seroprevalence of peste des petits ruminants (PPR) in Navsari and Valsad districts of South Gujarat. *International Journal of Current Microbiology and Applied Sciences* 2017; **6**: 221–228.
13. **Abubakar M, *et al.*** Evidence of peste des petits ruminants in serology of sheep and goats

from Sindh, Pakistan. *Transboundary and Emerging Diseases* 2011; **58**: 152–156.

14. **Zahur AB, *et al.*** Sero-epidemiology of peste des petits ruminants (PPR) in Pakistan. *Preventive Veterinary Medicine* 2011; **102**: 87–92.

15. **El-Yuguda AD, *et al.*** Seroprevalence of peste des petits ruminants among domestic small and large ruminants in the semi-arid region of north-eastern Nigeria. *Veterinary World* 2013; **6**: 807–811.

16. **Ishag O, Intisar K, Ali Y**. Detection of antibodies to peste des petits ruminants virus using passive haemagglutination test and cELISA in the White Nile state - Sudan, comparative study. *African Journal of Microbiology Research* 2014; **8**: 3475–3481.

17. **Kihu SM, *et al.*** Sero-epidemiology of peste des petits ruminants virus infection in Turkana county, Kenya. *BMC veterinary research* 2015; **11**: 1–14.

18. **Abubakar M, *et al.*** Peste des petits ruminants (PPR) in Pakistan: Analysis of a national level serological data. *Small Ruminant Research* 2017; **155**: 57–65.

19. **Dayhum A, *et al.*** Sero-prevalence and epidemiology of peste des petits ruminants in Libya. *Transboundary and Emerging Diseases* 2017; : 1–7.

20. **Saeed FA, Abdel-Aziz SA, Gumaa MM**. Seroprevalence and Associated Risk Factors of Peste des Petits Ruminants among Sheep and Goats in Kassala State, Sudan. *Open Journal of Animal Sciences* 2018; **8**: 381–395.

21. **Waret-Szkuta A, *et al.*** Peste des petits ruminants (PPR) in Ethiopia: Analysis of a national serological survey. *BMC Veterinary Research* 2008; **4**: 1–10.

22. **Kihu S, *et al.*** Appraisal of Peste des petits ruminants disease by Turkana pastoral community of Turkana county in Kenya. *American Journal of Research Communication* 2015; **2**: 1–29.

23. **Rony MS, *et al.*** Peste des petits ruminants risk factors and space – time clusters in Mymensingh, Bangladesh. *Transboundary and Emerging Diseases* 2017; **64**: 2042–2048.

24. **Bello AM, *et al.*** Research for peste des petits ruminants (PPR) virus antibodies in goats, sheep and gazelle from Bauchi and Gombe states, north eastern Nigeria. *Direct Research Journal of Agriculture and Food Science* 2016; **4**: 193–198.

25. **Parvez MA, Khatun R, Noman MA Al**. Prevalence and associated risk factors of peste des petits ruminants (PPR) in goat in Chittagong district, Bangladesh. *Research Journal for Veterinary Practitioners* 2014; **2**: 14–17.

26. **Thakor R, *et al.*** Seroprevalence of Peste des Petits Ruminants in Goats of South Gujarat. *Indian Journal of Small Ruminants* 2016; **22**: 25–254.

27. **El Arbi AS, *et al.*** Peste des petits ruminants virus, Mauritania. *Emerging Infectious Diseases* 2014; **20**: 333–336.

28. **Muse EA, *et al.*** Epidemiological investigation into the introduction and factors for spread of peste des petits ruminants, southern Tanzania. *Onderstepoort Journal of Veterinary Research* 2012; **79**: 2–7.

29. **Mbyuzi AO, *et al.*** Sero-prevalence and associated risk factors of peste des petits ruminants and contagious caprine pleuro-pneumonia in goats and sheep in the southern zone of Tanzania. *Preventive Veterinary Medicine* 2014; **116**: 138–144.

30. **Megersa B, *et al.*** Serological investigation of peste des petits ruminants (PPR) in small ruminants managed under pastoral and agro-pastoral systems in Ethiopia. *Small Ruminant Research* 2011; **97**: 134–138.

31. **Muse EA, Karimuribo ED, Misinzo G**. *Peste des petits ruminants (PPR) outbreak in southern Tanzania: Clinico-pathological findings, epidemiological investigation.*

Germany: Lambert Academic Publishing, 2013.

32. **Kardjadj M, Luka PD**. First serological and molecular evidence of PPRV occurrence in Ghardaïa district, center of Algeria. *Tropical Animal Health and Production* 2015; **47**: 1279–1284.

33. **Govindaraj G, Balamurugan V, Rahman H**. Estimation of economic loss of PPR in sheep and goats in India: An annual incidence based analysis. *British Journal of Virology* 2016; **3**: 77–85.

34. **Woma TY, *et al.*** Serosurvey of peste des petits ruminants virus in small ruminants from different agro-ecological zones of Nigeria. *The Onderstepoort Journal of Veterinary Research* 2016; **83**: 1–9.

35. **Rahman A, *et al.*** Short Communication: Serological detection of peste des petits ruminants virus (PPRV) in sheep and goats of Muzaffargarh district in South Punjab, Pakistan. *Veterinary Sciences: Research and Reviews* 2017; **2**: 82–88.

36. **Afera B, Hussien D, Amsalu K**. Seroprevalence of peste des petits ruminants in goats of southern parts of Tigray region. *Global Veterinaria* 2014; **12**: 512–516.

37. **Bari S, *et al.*** Hemato-biochemical parameters of Pesti-des Petits Ruminants (PPR) affected goats in Chittagong, Bangladesh. *Journal of Advanced Veterinary and Animal Research* 2018; **5**: 211–217.

38. **Faris D, *et al.*** Seroprevalence and sero-conversion after vaccination against peste des petits ruminants in sheep and goats from Awash Fentale District, Afar, Ethiopia. *Preventive Veterinary Medicine* 2012; **103**: 157–162.

39. **Mebrahtu K, *et al.*** Sero-epidemiological study of peste des petits ruminants (PPR) in sheep and goats under different production systems in South Omo, southern Ethiopia.

Small Ruminant Research 2018; **169**: 90–93.
